# Seasonal Dietary Intakes and Socioeconomic Status among Women in the Terai of Nepal

**Published:** 2014-06

**Authors:** Rebecca K. Campbell, Sameera A. Talegawkar, Parul Christian, Steven C. LeClerq, Subarna K. Khatry, Lee S.F. Wu, Keith P. West

**Affiliations:** ^1^Center for Human Nutrition, Department of International Health, Bloomberg School of Public Health, Johns Hopkins University, Baltimore, MD, USA; ^2^The Nepal Nutrition Intervention Project Sarlahi (NNIPS), National Society for the Prevention of Blindness, Kathmandu, Nepal

**Keywords:** Dietary adequacy, Female, Food frequency, Interviews, Rural population, Seasons, Socioeconomic factors, Undernutrition, Nepal

## Abstract

Despite widespread nutritional deficiencies, investigations of usual diet in rural South Asia remain sparse. The present study characterizes year-round and seasonal dietary patterns of women in the Terai of Nepal by sociodemographic status, using a novel, weekly single-visit and usual food frequency questionnaire that links recall to the agricultural season. The study was conducted across seasons in 2006-2008 among 15,899 women of reproductive age in Sarlahi district. Intakes were tabulated for all foods, overall and by socioeconomic status (SES), and in and out of season, as appropriate. Foods consumed regularly [median (interquartile range) weekly frequency] were rice [13 (7-13)], potatoes [10 (5-13)], legumes [6 (2-9)], and vegetable oil [13 (13-13)]. Animal products were infrequently consumed [1 (0-2) time per week] as were fruits and vegetables, most with a median weekly intake frequency of 0. Higher SES was associated with more frequent consumption of most food-groups, including in-season fruits and vegetables. Diets of women in the Terai of Nepal lack diversity and, likely, nutrient adequacy, which may pose health risks.

## INTRODUCTION

Undernutrition is a major cause of morbidity and mortality in low- and middle-income countries throughout the world and is estimated to affect 41% of the population of Nepal, with one of the highest rates in South Asia (1). Undernutrition in settings, like Nepal, may result from poor dietary quality and diversity, which can be attributed to limited food availability and access (2). Food availability in Nepal is constrained by population growth, low agricultural productivity, small land holdings, limited capital for farm improvements (3), and factors that inhibit access to food at the household and individual levels for poverty and food insecurity. Food insecurity is associated with poor health consequences in diverse settings and may be associated with increased risk of both undernutrition and chronic non-communicable diseases in low- and middle-income countries (4,5), a symptom of the widely-discussed “nutrition transition” (6).

Availability of and access to food in agrarian cultures lacking a strong market economy can be expected to be influenced by seasonality in temperature and precipitation and the resulting seasonal agricultural calendar. Seasonality affects the availability and pricing of foods as well as the daily workload and the burden of morbidity that can diminish nutritional status (3,7,8). Seasonality is a central characteristic of life in Nepal but its effects on diet sufficiency and health outcomes have not been explored. While micronutrient deficiencies have been shown to flux seasonally in pregnant women in Nepal (9), the degree to which this is driven by seasonal variation in diet quality remains unclear. Few dietary assessments in South Asia have considered seasonality in their survey design and analysis, and none has specifically reported the nature of seasonality in the diet or its effects on nutrient intake throughout the year (10-12). Renewed interest in diet quality through the lens of evolving nutrition transition and the need to characterize usual multiseason intakes in relation to chronic disease refocus attention on the need to assess the role of seasonality in dietary patterns in these settings.

In this paper, we present findings on dietary intake from an approximately 10-year follow-up assessment of a large cohort of women of reproductive age, who participated in a field trial on vitamin A or beta-carotene supplementation between 1993 and 1997. The study presents an opportunity to characterize the typical year-round and seasonal diets of women in an east-central Terai district of Nepal and examines variation in diet by locally-defined levels of socioeconomic status. The study also advances the potential to assess the usual intakes by adults in settings with highly seasonal dietary patterns with a single-administration questionnaire by incorporating information from the annual agricultural calendar to guide recall of intake frequencies for foods when in season.

## MATERIALS AND METHODS

### Setting

This dietary study was conducted in 2006-2008 among women of reproductive age living in Sarlahi district located in the southern plains (Terai) of Nepal. The subjects were members of a cohort that participated in a large maternal vitamin A or beta-carotene supplementation trial that took place from 1993 to 1997 (13). All women who participated in the original trial and continued to live in the study area at the time of follow-up were eligible to participate. The initial trial was approved by the Nepal Health Research Council (Kathmandu, Nepal), the Joint Committee on Clinical Investigation, Johns Hopkins School of Medicine (Baltimore, MD, USA), and the Teratology Society (Bethesda, MD, USA). The follow-up study was approved by the institutional review boards of the Johns Hopkins University and the Institute of Medicine, Tribhuvan University (Kathmandu, Nepal). Verbal consent was obtained from the women at the time of the follow-up interview.

### Dietary assessment

**Figure 1.**
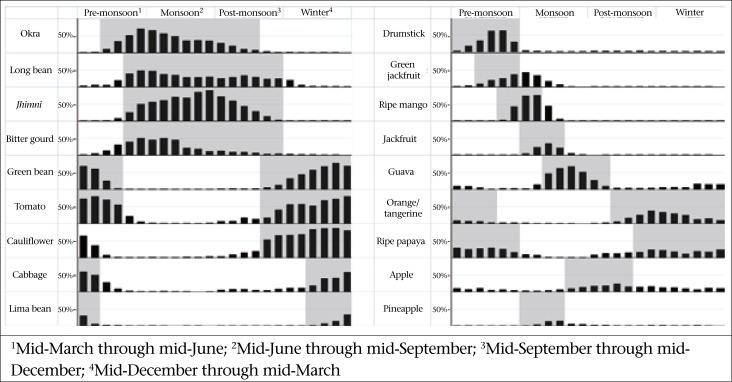
In-season periods and percentage of women consuming seasonal fruits and vegetables in the NNIPS-2 Cohort Follow-up Survey, Sarlahi, Nepal

An interviewer-administered food frequency questionnaire (FFQ) was developed specifically for this study. A total of 31 commonly-consumed foods were included in the questionnaire based on previous experience with dietary assessment in this population and focus group discussions conducted with female project staff (Table 1). Additionally, 28 fruits and vegetables separated into 10 ‘year-round foods’ that are available at a stable quantity and price throughout the year and 18 ‘seasonal foods’ that are available only during specific times of the year, based on data collected from the project staff who resided in the study area, were included in the questionnaire (Figure 1). Women were asked to report the usual frequency with which they consumed each food listed in the year preceding the interview, using day, week, month, or year as the unit. The same list of foods then was repeated, and the women were asked how many times they consumed each food in the seven days preceding the interview. In this analysis, only data from the past seven-day consumption frequencies were used.

### Assessment of covariates

During the follow-up study, trained interviewers visited each participant's house to collect data about her socioeconomic status, including household possessions, land ownership, physical quality of the house structure, the family's caste, and demographic characteristics, such as age, number of livebirths, level of education achieved, and literacy level. Mid-upper arm-circumference (MUAC), a reliable indicator of long-term nutritional status (14), was measured by the interviewers at the time of the home visit on the bare left arm, using a Ross insertion tape with standard protocol (15). Interviewers were specially trained in anthropometry procedures and were retrained periodically throughout the study to ensure standardization of measurements.

Indicators of socioeconomic status were selected for use in this analysis based on previously-published studies from the NNIPS-2 trial (16,17). Variables were dichotomized (yes/no) for possessions and house construction and simplified into categories for land ownership and caste. Demographic variables were also dichotomized or categorized. As has been done previously in this cohort, MUAC was dichotomized as greater than versus less than or equal to 21.5 cm (18).

### Analytical procedures

Prior to analysis, values above the 99th percentile of consumption frequency for each food item were recoded as missing and excluded from the analysis. A mean of 39.4 (of a sample of 15,899) was coded as missing per food, and no food had more than 233 outliers (1.5% of the study population). Individual outlying observations were dropped rather than excluding the participant from the analysis because food frequencies were not converted to total macro- or micronutrient consumption, and very few participants had outlying intake values for multiple foods. A small number of food items with similar nutrient profiles and consumption patterns were combined under a single name (e.g. fried snacks). Food-groups were classified according to those used in the Food Composition Table for Nepal 2012 (19). Some food-groups were combined and names changed accordingly in cases where small numbers of foods from each group were included in the questionnaire, and their consumption patterns were similar.

**Table 1.** Food frequency questionnaire items

Food-group	Name of food item (description)
Cereal and cereal products	Rice *bhat* (boiled rice)
Rice *roti* (flatbread)
Corn *dhirdo/bhat* (boiled corn)
Corn *roti* (flatbread)
Wheat *dhirdo/roti* (flatbread)
Millet *dhirdo/roti* (flatbread)
Pulses, legumes, and nuts	*Daal* (lentils)
*Maseura* (lentil patty)
Peanuts
Other legumes (include chickpeas, dried peas, and soybeans)
Vegetables	Green leafy vegetables
Dried green leafy vegetables
Eggplant
Green peas
*Lauka* (bottle gourd)
Ripe pumpkin
Green pumpkin
Green papaya
Tubers	Potatoes
Fruits	Banana
Meat, egg, and fish products	Chicken
Other meats (include goat, buff, and pig)
Large fish
Small fish
Snails
Eggs
Milk and milk products	Milk
Curd
Whey
Tea with milk
Fats and edible oil	Vegetable oil
*Ghyu* (clarified butter)
Hydrogenated oil
Miscellaneous	Noodles (packet)
Biscuits
*Samosas/pakaudas* (fried vegetable-filled snacks)
Beaten or puffed rice
Fried, sweet snacks
*Dalmot* (snack mix of fried rice, lentils, and spices)
Popped corn and whole roasted/boiled corn
Alcohol	*Jaard* (brewed millet drink)
Seasonal vegetables	Okra
Long bean
*Ghiraula, Jhimni* (sponge gourd)
Bitter gourd
Green bean
Tomato
Cauliflower
Cabbage
Lima bean
Drumstick
Green jackfruit
Seasonal fruits	Mango
Ripe jackfruit
Guava
Orange/tangerine
Ripe papaya
Apple
Pineapple

Some previously-assigned in-season periods were amended during analysis based on agricultural data contradicting the original seasonal delineations. Interviews conducted within the in-season period for each food were used in calculating the distribution of in-season consumption of that food, and interviews conducted not during the in-season period were used in measuring the out-of-season intake.

### Statistical analysis

Non-parametric [median (interquartile range—IQR)] summary statistics were calculated for the year-round and seasonal foods and, for seasonal foods, summary statistics were calculated separately by food for women who were interviewed during and outside of the in-season period. The Shapiro-Wilk test was used for checking the normality of the seasonal food distributions by season of the interview. As the intakes were not normally distributed, the Mann-Whitney Rank Sum test was used for assessing the equality of the distributions of intake in the in-season versus out-of-season periods. Median consumption frequencies of individual foods were examined numerically and graphically by month of interview to verify the constancy of intake of foods classified as year-round and the accuracy of the in-season periods assigned to seasonal foods. Median (IQR) intakes of individual foods and food-groups by level of key socioeconomic and nutritional status indicators were examined, and the non-parametric Mann-Whitney Rank Sum and Kruskal-Wallis equality of populations tests were used in comparing distributions of intake frequencies by level of covariates. All statistical analyses were performed using STATA (version 11.1) (StataCorp, College Station, TX).

## RESULTS

### Sociodemographic characteristics

A total of 16,320 women were recruited to participate in this study: 421 women were excluded from the present analysis because they reported being pregnant when the dietary questionnaire was administered, leaving a sample-size of 15,899. Most women were between 30 and 39 years of age (57.9%) (Table 2) and belonged to households that owned land (77.1%) and one or more cows or goats (85.7%). Only 19.5% of women lived in houses with cemented walls, a sign of wealthier status. The rate of literacy was 15.1%, and 28.9% were classified as thin based on mid-upper arm-circumference ≤21.5 cm. Socioeconomic status of the studied women was comparable across seasons of interview (data not shown).

**Table 2.** Sociodemographic characteristics of women in the NNIPS-2 Cohort Follow-up Survey (2006-2008), Sarlahi, Nepal

Age (n=15,578)	%
<30	25.7
30-39	57.9
>40	16.4
MUAC ≤21.5 cm (n=15,869)	28.9
Livebirths (n=15,812)	
≤2	8.6
3-6	72.6
>6	18.8
Literate (n=15,878)	15.1
Attended school (n=15,876)	14.5
Caste (n=15,879)	
Brahmin	6.8
Chhetri	6.8
Vaiysha	66.2
Shudra	12.2
Non-Hindu*	7.9
Ownership of land (n=15,800)	
1-9 *kattha*†	33.0
≥10 *kattha*†	44.1
Livestock (n=15,873)	85.7
Cart (n=15,873)	12.5
House construction	
Cement walls (n=15,877)	19.5
Cement roof (n=15,878)	5.0
Upper floor (n=15,875)	49.1

*Includes Muslim, Buddhist, and Christian;

†1 *kattha*=0.034 hectares or 0.084 acres

### Year-round foods

**Figure 2.**
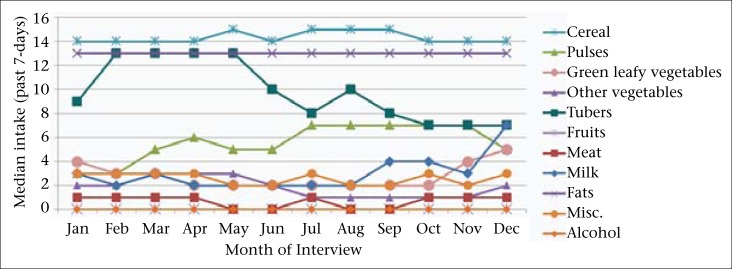
Women's median consumption of year-round food-groups by month of interview in the NNIPS-2 Cohort Follow-up Survey, Sarlahi, Nepal

Figure 2 reveals that the median weekly intakes of foods categorized *apriori* as available year-round were stable throughout the year as reported by women interviewed across different calendar months. A uniform intake frequency was evident for some food-groups while others, such as pulses, legumes and nuts, green leafy vegetables, and other year-round vegetables and tubers displayed some seasonal variation throughout the year.

Among the year-round foods, cereal and cereal products were reportedly consumed a median of 14 (IQR 13-17) times per week, and, among individual items, rice was most frequently eaten [13 (7-13) times], followed by wheat [2 (0-6) times]. Among fats and edible oil eaten 13 (13-14) times per week, vegetable oil was most often consumed [13 (13-13) times], with <25% of the subjects reporting to have eaten local clarified butter (*ghyu*) and hydrogenated oils [0 (0-0) times per week for both] in the previous week (Table 3). Potatoes and pulses, legumes and nuts were often consumed with median intakes of 10 (5-13) and 6 (2-9) times respectively during the preceding week. On the other hand, foods of animal source, such as milk and milk products, were far less consumed [3 (0-9) times in the previous week], with one-fourth of the study population reporting eating none. Foods from the meat, egg, and fish products group were infrequently eaten, 1 (0-2) time per week, with median intake of individual meats being zero. Year-round vegetables were reportedly eaten 4 (2-7) times per week, with green leafy vegetables accounting for half [2 (0-4)] of those consumed. When food-groups were disaggregated, most individual foods investigated had median intakes of zero, with only small percentages of study subjects consuming any. The minimum intake of each year-round food-group was also zero, consistent with a minute fraction of all respondents (<0.1%) not consuming foods in each group the previous week.

### Seasonal foods

**Figure 3.**
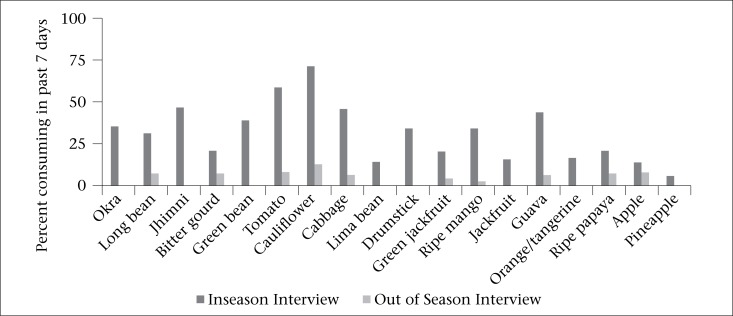
Percentage of women consuming seasonal foods 1+ times in the past seven days by timing of interview relative to seasonality of foods in the NNIPS-2 Cohort Follow-up Survey, Sarlahi, Nepal

Consumption of most seasonal fruits and vegetables was low, even in season (Table 4). Women regularly consumed only tomato and cauliflower when in season, reflected by a median intake frequency of twice weekly for each (IQR: 0-7 and 0-5 respectively). Minimum and maximum frequencies of intake were comparable in and out-of-season for listed vegetables and fruits, with seasonal variation driven almost entirely by differences across the 75th to 95th percentiles (Mann-Whitney tests of equality of food-specific seasonal distributions, p<0.0001). For all seasonal vegetables and fruits, more women reported consuming each at least once during an in-season interview compared to interviews that were conducted out-of-season, with differences being statistically significant (Figure 3).

### Socioeconomic status (SES) and diet

Most foods, whether available year-round or seasonally, were eaten more often by women of higher socioeconomic standing, reflected by caste, literacy, land and livestock ownership, house construction, and size of arm-circumference (Table 5). Exceptions were cereal and cereal products and fats and edible oils, which were regularly consumed (13-14 times per week) across the socioeconomic gradients, and meat, egg, and fish products, which were rarely consumed (~1 time per week), irrespective of SES. Consumption of milk and milk products especially varied by SES, with literate women consuming milk items a median of 5 (IQR 1-13) versus 0 (0-2) times among non-literate respondents. Women living in houses with cemented walls and in lower-quality houses reportedly consumed milk products 7 (2-14) times versus 2 (0-8) times in the previous week respectively. Intake of pulses, legumes and nuts, tubers, and miscellaneous foods also tracked SES. Table 6 (A-F) shows that some individual foods, such as dried soybean balls (*maseura*) and corn, were more frequently consumed by women of lower means, with differences being statistically significant for most SES indicators.

## DISCUSSION

Our findings indicate that, while rural women in the poor, central Terai region of Nepal were consuming staple foods, such as rice, potatoes, *daal* (lentils), and vegetable oil twice daily, irrespective of wealth; intakes of vegetables, fruits, and animal products were far less frequent, especially among women of lower socioeconomic means. Intake frequencies of seasonal foods rose in season but mostly among women of higher socioeconomic status. Women of lower socioeconomic status consumed less pulses, legumes and nuts, milk and milk products, tubers, year-round fruits and vegetables, and miscellaneous snacks than women of higher means. Very low intakes of dietary varieties and infrequent vegetable, fruit, and meat have been observed previously in the Terai and elsewhere in rural Nepal and northern India (20-22) and are likely responsible for observed insufficient micronutrient intakes and deficiencies in those settings (22,23). In this population, more than 50% of women were thin by arm-circumference assessment (≤21.5 cm), reflecting a chronic state of wasting malnutrition (13). Our findings agree with those from rural Bangladesh where intakes of non-staple and animal-source foods are positively associated with SES (24,25).

**Table 3.** Seven-day food intake frequencies of year-round foods by women in NNIPS-2 Cohort Follow-up Survey, Sarlahi, Nepal (n=15,858)*†

Food/Food-group	Minimum	25th percentile	Median	75th percentile	95th percentile	Maximum
Cereal and cereal products	0	13	14	17	21	38
Rice‡	0	7	13	13	15	25
Corn¶	0	0	0	1	7	20
Wheat *roti*	0	0	2	6	7	12
Millet *roti*	0	0	0	0	1	7
Pulses, legumes, and nuts	0	2	6	9	15	29
Daal	0	2	3	7	13	13
Maseura	0	0	0	2	4	12
Peanut	0	0	0	0	2	7
Other legumes	0	0	0	1	3	7
Vegetables	0	2	4	7	13	31
Leafy green vegetables	0	0	2	4	10	13
Dried leafy green vegetables	0	0	0	0	3	7
Eggplant	0	0	0	1	3	10
Green peas	0	0	0	0	1	4
Gourd	0	0	0	1	3	6
Ripe pumpkin	0	0	0	0	1	3
Green pumpkin	0	0	0	0	0	2
Green papaya	0	0	0	0	1	3
Tubers	0	5	10	13	13	14
Potatoes	0	5	10	13	13	14
Fruits	0	0	0	1	2	5
Banana	0	0	0	1	2	5
Meat, egg, and fish products	0	0	1	2	5	13
Chicken	0	0	0	0	1	3
Other meat	0	0	0	1	2	5
Fish	0	0	0	1	2	6
Snails	0	0	0	0	1	2
Eggs	0	0	0	0	1	4
Milk and milk products	0	0	3	9	21	42
Milk	0	0	1	6	13	14
Curd	0	0	0	1	6	7
Whey	0	0	0	0	2	7
Tea	0	0	0	2	13	14
Fats and edible oil	0	13	13	14	20	29
Vegetable oil	0	13	13	13	14	14
Ghyu	0	0	0	0	7	13
Hydrogenated oil	0	0	0	0	1	4
Miscellaneous	0	1	3	5	10	24
Fried snacks§	0	0	0	2	4	13
Biscuits	0	0	0	0	2	5
Unfried snacks**	0	0	2	3	7	14
Alcohol	0	0	0	0	2	13
Jaard	0	0	0	0	2	13

*Missing values ranged from 0 to 187 for individual food items, except for fried snacks which was missing 462 values;

†Outliers above the 99th percentile were excluded for each food to avoid extreme and unlikely values;

‡Includes boiled rice and rice-flour bread;

¶Includes boiled corn and corn-flour bread;

§Includes noodles, *samosas*, *pakaudas*, fried sweet snacks, and *dalmot*;

**Includes unfried puffed or roasted corn and rice snacks

Although median intake frequencies of in-season foods rose among women of higher socioeconomic status, intakes of seasonal fruits and vegetables were, nonetheless, extremely low, with most usual intake frequencies remaining nil. Seasonally, higher intakes were only evident among most-frequent consumers, evident only at or above the 75th percentiles of intake. The sole exceptions were tomato and cauliflower intakes, which rose from median intake of zero to two times between out-of-season and in-season periods.

In a setting where livelihood is largely dependent on agriculture, one might reasonably expect a direct, positive relationship between seasonal production and dietary intake. Yet, seasonal variation in diet was largely blunted in this Terai population. Multiple economic factors may be responsible for this. A large proportion of the Terai population in Nepal owns insufficient land for subsistence, requiring the poor to purchase food (26). As such, most food expenditure is made on staple foods, with little remaining for purchasing fruits, vegetables, and animal-source foods (26). Households supported by day-labour or tilling rented land or borrowing money to cope with shocks (e.g. prolonged illness or food insecurity) are also common in this setting (26,27) and may not experience seasonal fluctuation in disposable income. While market availability and pricing of seasonal produce may fluctuate throughout the year, the food expenditure of the poorest segments of the population may not allow for incorporating those foods in the usual diet. Further, much remains to be described about the supply side of local markets, including food availability, variety, and pricing. Our findings highlight the inaccessibility of a diverse, nutritionally-adequate diet in this population and reinforce the existence of major gaps in knowledge about the agro-economic dynamics that limit access to diverse food baskets in rural Nepal (28).

### Strengths and limitations

Strengths of the present study include its large sample-size, the use of an instrument prompting recall of 7 days of intake, and a novel attempt to separate, in one assessment, seasonal from year-round food intake patterns. Limitations of the study include the absence of data about portion-size and reliance on the data on local farmers and resident focus groups rather than measured agricultural data to define in-season periods for foods. Nonetheless, we found interrelationships in expected directions between reported dietary intake patterns, arm-circumference, reflecting wasting and generalized nutritional stress, and socioeconomic status, supporting the plausibility of our food frequency distributions.

**Table 4.** Weekly intake frequencies among women for seasonal foods by timing of interview relative to in-season period of each food in NNIPS-2 Cohort Follow-up Survey, Sarlahi, Nepal*

Food	In-season†							Out-of-season						
	n‡	Minimum	25th percentile	Median	75th percentile	95th percentile	Maximum	n	Minimum	25th percentile	Median	75th percentile	95th percentile	Maximum
Vegetables														
Okra	8,818	0	0	0	1	4	7	6,998	0	0	0	0	0	7
Long bean	8,900	0	0	0	1	3	6	6,816	0	0	0	0	1	6
*Jhimni*	8,874	0	0	0	2	6	8	6,858	0	0	0	0	0	7
Bitter gourd	8,896	0	0	0	0	2	3	6,840	0	0	0	0	1	3
Green bean	8,403	0	0	0	2	4	8	7,317	0	0	0	0	0	7
Tomato	8,412	0	0	2	7	13	13	7,311	0	0	0	0	2	13
Cauliflower	5,177	0	0	2	5	13	13	10,651	0	0	0	0	2	13
Cabbage	3,415	0	0	0	2	4	5	12,300	0	0	0	0	1	5
Lima bean	3,427	0	0	0	0	2	2	12,305	0	0	0	0	0	2
Drumstick	4,906	0	0	0	1	3	4	10,851	0	0	0	0	0	4
Green jackfruit	6,099	0	0	0	0	2	2	9,626	0	0	0	0	0	2
Fruits														
Ripe mango	2,303	0	0	0	1	4	6	13,411	0	0	0	0	0	6
Jackfruit	1,659	0	0	0	0	1	1	14,062	0	0	0	0	0	1
Guava	3,379	0	0	0	2	7	7	12,422	0	0	0	0	1	7
Orange/tangerine	9,799	0	0	0	0	2	4	5,979	0	0	0	0	0	4
Ripe papaya	10,092	0	0	0	0	2	4	5,635	0	0	0	0	1	4
Apple	3,628	0	0	0	0	2	3	12,084	0	0	0	0	1	3
Pineapple	1,732	0	0	0	0	1	1	14,024	0	0	0	0	0	1

*p<0.0001 for Mann-Whitney Rank Sum tests of equality of distribution of intake by season of interview for all foods;

‡In-season and out-of-season periods based on seasons defined in Figure 1;

‡Number of women interviewed during in-season period for each food item

### Conclusions

The present study reveals starkly inadequate year-round and seasonal dietary intakes among adult women in the Terai region of Nepal. While staple foods and accompaniments were usually eaten twice daily, intakes of vegetables, fruits, and animal-source foods were, with a few exceptions, very infrequent, especially among the poorer members of this rural society. These dietary patterns are consistent with chronic wasting malnutrition (29) and multiple micronutrient deficiencies (9,29) that have been repeatedly observed in this region of Nepal. Future studies are needed to examine relationships between seasonal agricultural production, market availability and price, dietary intakes and their causal pathways, and the effects of these factors on nutritional status of rural populations.

### Key messages

- Diet variety is extremely low among women in the Terai of Nepal
- Intakes of fruit, vegetables, and animal products are particularly low
- Poorer women cannot avail themselves of even seasonally-available foods
- Women of higher socioeconomic means consume more seasonal fruits and vegetables only during their in-season periods.

## ACKNOWLEDGEMENTS

We thank Christine Stewart, Joanne Katz, James Tielsch, Luke Mullany, Sharada Ram Shrestha (deceased), Darrell Mast, Andre Hackman, Tirta Raj Sakya, and field and data management staff of the study team.

This study was supported by Grant no. GH614 (Control of Global Micronutrient Deficiency) between the Bill & Melinda Gates Foundation, Seattle, and the Center for Human Nutrition, Department of International Health of the Johns Hopkins Bloomberg School of Public Health, Baltimore and was undertaken in collaboration with the National Society for the Prevention of Blindness (Nepal Netra Jyoti Sangh), Kathmandu, Nepal. The original vitamin A supplementation trial was carried out under Cooperative Agreement no. DAN 0045-A-5094 between the Office of Nutrition, US Agency for International Development, Washington, DC and the Johns Hopkins University, with additional assistance from the Sight and Life Research Institute, Baltimore, MD. The funding agencies had no role in the study design, data collection, data analysis, data interpretation, or writing of the report.

**Table 5.** Food-group consumption patterns among women presented as median (25th percentile–75th percentile) intake frequencies during the past seven days by socioeconomic and nutritional status, NNIPS-2 Cohort Follow-up Survey, Sarlahi, Nepal*†

Characterisitics	Cereal and cereal products	Pulses, legumes, and nuts	Vegetables (year-round)	Tubers	Fruits (year-round)	Meat, egg, and fish products	Milk and milk products	Fats and edible oil	Miscellaneous	Alcohol
Caste																				
Hindu-High‡	14 (13,17)	8 (4,12)	6 (3,9)	13 (7,13)	0 (0,1)	1 (0,2)	14 (7,21)	14 (13,16)	5 (2,8)	0 (0,0)
Hindu-Low¶	14 (13,17)	5 (2,8)	4 (2,7)	10 (5,13)	0 (0,1)	1 (0,2)	2 (0,8)	13 (13,14)	2 (1,5)	0 (0,0)
Non-Hindu§	14 (13,18)	5 (2,8)	3 (1,5)	11 (7,13)	0 (0,0)	2 (1,3)	2 (0,7)	13 (13,14)	2 (1,4)	0 (0,0)
Literate										
Yes	14 (13,17)	8 (6,13)	6 (3,9)	3 (7,13)	0 (0,1)	1 (0,2)	5 (1,13)	14 (13,16)	5 (2,8)	0 (0,0)
No	14 (13,17)	5 (2,8)	4 (2,7)	2 (5,13)	0 (0,0)	1 (0,2)	0 (0,2)	13 (13,13)	2 (1,5)	0 (0,0)
Land in kattha**																				
≥10	14 (13,18)	7 (4,10)	5 (2,8)	11 (6,13)	0 (0,1)	1 (0,2)	7 (1,14)	13 (13,15)	3 (1,6)	0 (0,0)
1-9	14 (13,17)	5 (2,8)	4 (2,7)	10 (5,13)	0 (0,0)	1 (0,2)	2 (0,8)	13 (13,13)	2 (1,5)	0 (0,0)
None	14 (13,16)	4 (2,7)	3 (2,6)	10 (5,13)	0 (0,0)	1 (0,2)	1 (0,5)	13 (13,13)	2 (0,4)	0 (0,0)
Livestock																				
≥1	14 (13,18)	6 (3,9)	4 (2,7)	10 (5,13)	0 (0,1)	1 (0,2)	3 (0,10)	13 (13,14)	3 (1,5)	0 (0,0)
None	14 (13,16)	5 (2,8)	4 (2,6)	11 (5,13)	0 (0,0)	1 (0,2)	2 (0,7)	13 (13,13)	3 (1,5)	0 (0,0)
Cemented walls																				
Yes	14 (13,18)	7 (4,11)	4 (2,7)	13 (7,13)	0 (0,1)	1 (0,2)	7 (2,14)	13 (13,15)	3 (1,7)	0 (0,0)
No	14 (13,17)	5 (2,8)	4 (2,7)	10 (5,13)	0 (0,0)	1 (0,2)	2 (0,8)	13 (13,14)	2 (1,5)	0 (0,0)
MUAC (cm)																				
>21.5	14 (13,17)	6 (3,9)	4 (2,7)	10 (6,13)	0 (0,1)	1 (0,2)	3 (0,11)	13 (13,14)	3 (1,6)	0 (0,0)
≤21.5	14 (13,17)	5 (2,8)	4 (2,7)	10 (5,13)	0 (0,0)	1 (0,2)	2 (0,7)	13 (13,13)	2 (0,4)	0 (0,0)

*Wilcoxon Rank Sum test was used for comparing distributions by measures with two categories (livestock ownership, cemented walls, MUAC, literacy). Kruskal-Wallis Rank test was used for measures with three categories (caste, land ownership). All comparisons were statistically significant at p≤0.0001, except cereal and cereal products by caste, MUAC, and literacy; miscellaneous, alcohol, tubers, and fruits by livestock ownership; and year-round vegetables by cemented walls. Cereal consumption by MUAC was statistically significant at p<0.05;

†n=14,838-15,092, except miscellaneous group by caste, where n=13,365;

‡Includes Brahmin and Chhetri;

¶Includes Vaiysha and Shudra;

§Includes Muslim, Buddhist, and Christian;

**1 *kattha*=0.034 hectares or 0.084 acres

**Table 6 (A-F).** Consumption patterns by women's sociodemographic and nutritional status presented as median (25th percentile-75th percentile intake of individual foods) in the past seven days by NNIPS-2 Cohort Follow-up Survey, Sarlahi, Nepal

A. Year-round foods: cereal, pulses, and milk products												
Characteristics	Cereal and cereal products				Pulses, legumes, and nuts				Milk and milk products			
	Rice	Corn	Wheat	Millet	*Daal*	*Maseura*	Peanuts	Other	Milk	Curd	Whey	Tea
Caste												
Hindu-High	13 (9,13)	0 (0,1)	0 (0,6)	0 (0,0)	7 (2,7)	0 (0,1)	0 (0,0)	0 (0,2)	7 (0,9)	1 (0,3)	0 (0,2)	7 (0,7)
Hindu-Low	13 (7,13)	0 (0,1)	2 (0,7)	0 (0,0)	3 (1,7)	0 (0,2)	0 (0,0)	0 (0,0)	0 (0,4)	0 (0,1)	0 (0,0)	0 (0,0)
Non-Hindu	13 (8,13)	0 (0,0)	2 (0,7)	0 (0,0)	4 (2,7)	0 (0,1)	0 (0,0)	0 (0,0)	0 (0,3)	0 (0,1)	0 (0,0)	0 (0,1)
Land in *kattha*												
≥10	13 (7,13)	0 (0,1)	2 (0,7)	0 (0,0)	6 (2,7)	0 (0,1)	0 (0,0)	0 (0,1)	2 (0,7)	0 (0,2)	0 (0,0)	0 (0,7)
1-9	13 (7,13)	0 (0,2)	0 (0,6)	0 (0,0)	3 (1,7)	0 (0,2)	0 (0,0)	0 (0,0)	0 (0,3)	0 (0,1)	0 (0,0)	0 (0,1)
None	13 (7,13)	0 (0,0)	1 (0,6)	0 (0,0)	2 (1,6)	0 (0,2)	0 (0,0)	0 (0,0)	0 (0,2)	0 (0,1)	0 (0,0)	0 (0,0)
Livestock												
≥1	13 (7,13)	0 (0,1)	2 (0,6)	0 (0,0)	4 (2,7)	0 (0,2)	0 (0,0)	0 (0,1)	1 (0,7)	0 (0,1)	0 (0,0)	0 (0,2)
None	13 (7,13)	0 (0,0)	1 (0,6)	0 (0,0)	3 (1,7)	0 (0,1)	0 (0,0)	0 (0,0)	0 (0,2)	0 (0,1)	0 (0,0)	0 (0,3)
Cemented walls												
Yes	13 (7,13)	0 (0,0)	5 (0,7)	0 (0,0)	7 (3,7)	0 (0,1)	0 (0,0)	0 (0,1)	2 (0,7)	0 (0,2)	0 (0,0)	0 (0,7)
No	13 (7,13)	0 (0,1)	1 (0,6)	0 (0,0)	3 (1,7)	0 (0,2)	0 (0,0)	0 (0,0)	0 (0,4)	0 (0,1)	0 (0,0)	0 (0,1)
MUAC (cm)												
>21.5	13 (7,13)	0 (0,1)	1 (0,6)	0 (0,0)	4 (2,7)	0 (0,1)	0 (0,0)	0 (0,1)	1 (0,7)	0 (0,1)	0 (0,0)	0 (0,4)
≤21.5	13 (7,13)	0 (0,0)	2 (0,7)	0 (0,0)	3 (1,7)	0 (0,2)	0 (0,0)	0 (0,0)	0 (0,3)	0 (0,1)	0 (0,0)	0 (0,0)
Literate												
Yes	13 (8,13)	0 (0,1)	1 (0,6)	0 (0,0)	7 (4,7)	0 (0,1)	0 (0,0)	0 (0,2)	4 (0,7)	0 (0,2)	0 (0,1)	7 (0,7)
No	13 (7,13)	0 (0,1)	2 (0,6)	0 (0,0)	3 (1,7)	0 (0,2)	0 (0,0)	0 (0,0)	0 (0,4)	0 (0,1)	0 (0,0)	0 (0,0)

B. Year-round foods: vegetables, tubers, and fruits										
Characteristics	Vegetables								Tubers	Fruits
	Green leafy vegetables	Dried green leafy vegetables	Eggplant	Peas	Bitter gourd	Ripe pumpkin	Green pumpkin	Green papaya	Potatoes	Banana
Caste										
Hindu-High	3 (2,7)	0 (0,2)	0 (0,0)	0 (0,0)	0 (0,0)	0 (0,0)	0 (0,0)	0 (0,0)	13 (7,13)	0 (0,1)
Hindu-Low	2 (0,3)	0 (0,0)	0 (0,1)	0 (0,0)	0 (0,1)	0 (0,0)	0 (0,0)	0 (0,0)	10 (5,13)	0 (0,1)
Non-Hindu	1 (0,2)	0 (0,0)	0 (0,1)	0 (0,0)	0 (0,1)	0 (0,0)	0 (0,0)	0 (0,0)	11 (7,13)	0 (0,0)
Land in *kattha*										
≥10	2 (1,4)	0 (0,0)	0 (0,1)	0 (0,0)	0 (0,1)	0 (0,0)	0 (0,0)	0 (0,0)	11 (6,13)	0 (0,1)
1-9	2 (0,4)	0 (0,1)	0 (0, 1)	0 (0,0)	0 (0,1)	0 (0,0)	0 (0,0)	0 (0,0)	10 (5,13)	0 (0,0)
None	2 (0, 3)	0 (0,0)	0 (0,1)	0 (0,0)	0 (0,1)	0 (0,0)	0 (0,0)	0 (0,0)	10 (5,13)	0 (0,0)
Livestock										
≥1	2 (0,4)	0 (0,0)	0 (0,1)	0 (0,0)	0 (0,1)	0 (0,0)	0 (0,0)	0 (0,0)	10 (5,13)	0 (0,1)
None	2 (0,3)	0 (0,0)	0 (0,1)	0 (0,0)	0 (0,1)	0 (0,0)	0 (0,0)	0 (0,0)	11 (5,13)	0 (0,0)
Cemented walls										
Yes	2 (1,4)	0 (0,0)	0 (0,1)	0 (0,0)	0 (0,1)	0 (0,0)	0 (0,0)	0 (0,0)	13 (7,13)	0 (0,1)
No	2 (0,4)	0 (0,0)	0 (0,1)	0 (0,0)	0 (0,1)	0 (0,0)	0 (0,0)	0 (0,0)	10 (5,13)	0 (0,0)
MUAC (cm)										
>21.5	2 (1,4)	0 (0,1)	0 (0,1)	0 (0,0)	0 (0,1)	0 (0,0)	0 (0,0)	0 (0,0)	10 (6,13)	0 (0,1)
≤21.5	2 (0,3)	0 (0,0)	0 (0,1)	0 (0,0)	0 (0,1)	0 (0,0)	0 (0,0)	0 (0,0)	10 (5,13)	0 (0,0)
Literate										
Yes	3 (1,6)	0 (0,1)	0 (0,1)	0 (0,0)	0 (0,1)	0 (0,0)	0 (0,0)	0 (0,0)	13 (7,13)	0 (0,1)
No	2 (0,3)	0 (0,0)	0 (0,1)	0 (0,0)	0 (0,1)	0 (0,0)	0 (0,0)	0 (0,0)	10 (5,13)	0 (0,0)

C. Year-round foods: meat, fats, miscellaneous foods, and alcohol												
Characteristics	Meat, egg, and fish products			Fats and edible oil			Miscellaneous			Alcohol
	Chicken	Other meat	Fish	Snails	Eggs	Vegetable oil	*Ghyu*	Hydrogenated oil	Fried snacks	Biscuits	Unfried snacks	*Jaard*
Caste												
Hindu-High	0 (0,0)	0 (0,1)	0 (0,0)	0 (0,0)	0 (0,0)	13 (13,13)	1 (0,3)	0 (0,0)	0 (0,2)	0 (0,0)	3 (1,7)	0 (0,0)
Hindu-Low	0 (0,0)	0 (0,1)	0 (0,1)	0 (0,0)	0 (0,0)	13 (13,13)	0 (0,0)	0 (0,0)	0 (0,2)	0 (0,0)	2 (0,3)	0 (0,0)
Non-Hindu	0 (0,1)	1 (0,1)	0 (0,1)	0 (0,0)	0 (0,1)	13 (13,13)	0 (0,0)	0 (0,0)	1 (0,2)	0 (0,0)	1 (0,3)	0 (0,0)
Land in *kattha*												
≥10	0 (0,0)	0 (0,1)	0 (0,0)	0 (0,0)	0 (0,0)	13 (13,13)	0 (0,1)	0 (0,0)	0 (0,2)	0 (0,0)	2 (0,4)	0 (0,0)
1-9	0 (0,0)	0 (0,1)	0 (0,1)	0 (0,0)	0 (0,0)	13 (13,13)	0 (0,0)	0 (0,0)	0 (0,1)	0 (0,0)	2 (0,3)	0 (0,0)
None	0 (0,0)	0 (0,1)	0 (0,1)	0 (0,0)	0 (0,0)	13 (13,13)	0 (0,0)	0 (0,0)	0 (0,1)	0 (0,0)	1 (0,3)	0 (0,0)
Livestock												
≥1	0 (0,0)	0 (0,1)	0 (0,0)	0 (0,0)	0 (0,0)	13 (13,13)	0 (0,0)	0 (0,0)	0 (0,2)	0 (0,0)	2 (0,4)	0 (0,0)
None	0 (0,0)	0 (0,1)	0 (0,1)	0 (0,0)	0 (0,0)	13 (13,13)	0 (0,0)	0 (0,0)	1 (0,2)	0 (0,0)	2 (0,3)	0 (0,0)
Cemented walls												
Yes	0 (0,0)	0 (0,1)	0 (0,1)	0 (0,0)	0 (0,0)	13 (13,13)	0 (0,1)	0 (0,0)	1 (0,2)	0 (0,0)	2 (0,4)	0 (0,0)
No	0 (0,0)	0 (0,1)	0 (0,1)	0 (0,0)	0 (0,0)	13 (13,13)	0 (0,0)	0 (0,0)	0 (0,1)	0 (0,0)	2 (0,3)	0 (0,0)
MUAC (cm)												
>21.5	0 (0,0)	0 (0,1)	0 (0,0)	0 (0,0)	0 (0,0)	13 (13,13)	0 (0,0)	0 (0,0)	0 (0,2)	0 (0,0)	2 (0,4)	0 (0,0)
≤21.5	0 (0,0)	0 (0,0)	0 (0,1)	0 (0,0)	0 (0,0)	13 (13,13)	0 (0,0)	0 (0,0)	0 (0,1)	0 (0,0)	1 (0,3)	0 (0,0)
Literate												
Yes	0 (0,0)	0 (0,1)	0 (0,0)	0 (0,0)	0 (0,0)	13 (13,13)	0 (0,3)	0 (0,0)	1 (0,3)	0 (0,1)	3 (1,7)	0 (0,0)
No	0 (0,0)	0 (0,1)	0 (0,1)	0 (0,0)	0 (0,0)	13 (13,13)	0 (0,0)	0 (0,0)	0 (0,1)	0 (0,0)	2 (0,3)	0 (0,0)

D. Seasonal vegetables during their in-season periods											
Characteristics	Seasonal vegetables										
	Okra	Long bean	*Jhimni*	Bitter gourd	Green bean	Tomato	Cauliflower	Cabbage	Lima bean	Drumstick	Green jackfruit
Caste											
Hindu-High	0 (0,2)	0 (0,2)	0 (0,2)	0 (0,0)	0 (0,2)	7 (1,13)	2 (0,4)	1 (0,2)	0 (0,0)	0 (0,0)	0 (0,0)
Hindu-Low	0 (0,1)	0 (0,1)	0 (0,2)	0 (0,0)	0 (0,2)	1 (0,6)	2 (0,5)	0 (0,2)	0 (0,0)	0 (0,1)	0 (0,0)
Non-Hindu	0 (0,1)	0 (0,1)	0 (0,2)	0 (0,0)	0 (0,2)	1 (0,3)	3 (1,6)	0 (0,2)	0 (0,0)	0 (0,0)	0 (0,0)
Land in *kattha*											
≥10	0 (0,2)	0 (0,1)	0 (0,3)	0 (0,0)	0 (0,2)	2 (0,7)	2 (0,5)	0 (0,2)	0 (0,0)	0 (0,1)	0 (0,0)
1-9	0 (0,1)	0 (0,1)	0 (0,2)	0 (0,0)	0 (0,2)	2 (0,7)	2 (0,5)	0 (0,2)	0 (0,0)	0 (0,1)	0 (0,0)
None	0 (0,1)	0 (0,1)	0 (0,2)	0 (0,0)	0 (0,2)	1 (0,5)	3 (0,5)	0 (0,2)	0 (0,0)	0 (0,1)	0 (0,0)
Livestock											
≥1	0 (0,1)	0 (0,1)	0 (0,2)	0 (0,0)	0 (0,2)	2 (0,7)	2 (0,5)	0 (0,2)	0 (0,0)	0 (0,1)	0 (0,0)
None	0 (0,1)	0 (0,1)	0 (0,2)	0 (0,0)	0 (0,2)	2 (0,7)	3 (1,6)	0 (0,2)	0 (0,0)	0 (0,1)	0 (0,0)
Cemented walls											
Yes	0 (0,2)	0 (0,1)	1 (0,3)	0 (0,1)	0 (0,2)	3 (0,10)	3 (1,6)	1 (0,2)	0 (0,0)	0 (0,1)	0 (0,0)
No	0 (0,1)	0 (0,1)	0 (0,2)	0 (0,0)	0 (0,2)	2 (0,7)	2 (0,5)	0 (0,2)	0 (0,0)	0 (0,1)	0 (0,0)
MUAC (cm)											
>21.5	0 (0,1)	0 (0,1)	0 (0,2)	0 (0,0)	0 (0,2)	2 (0,8)	2 (0,5)	0 (0,2)	0 (0,0)	0 (0,1)	0 (0,0)
≤21.5	0 (0,1)	0 (0,1)	0 (0,2)	0 (0,0)	0 (0,1)	0 (0,3)	2 (0,4)	0 (0,2)	0 (0,0)	0 (0,1)	0 (0,0)
Literate											
Yes	0 (0,2)	0 (0,2)	0 (0,3)	0 (0,0)	0 (0,2)	7 (1,13)	3 (1,6)	1 (0,2)	0 (0,0)	0 (0,1)	0 (0,1)
No	0 (0,1)	0 (0,1)	0 (0,2)	0 (0,0)	0 (0,2)	1 (0,6)	2 (0,5)	0 (0,2)	0 (0,0)	0 (0,1)	0 (0,0)

E. Seasonal vegetables during their out-of-season periods											
Characteristics	Seasonal vegetables										
	Okra	Long bean	*Jhimni*	Bitter gourd	Green bean	Tomato	Cauliflower	Cabbage	Lima bean	Drumstick	Green jackfruit
Caste											
Hindu-High	0 (0,0)	0 (0,0)	0 (0,0)	0 (0,0)	0 (0,0)	0 (0,0)	0 (0,0)	0 (0,0)	0 (0,0)	0 (0,0)	0 (0,0)
Hindu-Low	0 (0,0)	0 (0,0)	0 (0,0)	0 (0,0)	0 (0,0)	0 (0,0)	0 (0,0)	0 (0,0)	0 (0,0)	0 (0,0)	0 (0,0)
Non-Hindu	0 (0,0)	0 (0,0)	0 (0,0)	0 (0,0)	0 (0,0)	0 (0,0)	0 (0,0)	0 (0,0)	0 (0,0)	0 (0,0)	0 (0,0)
Land in *kattha*											
≥10	0 (0,0)	0 (0,0)	0 (0,0)	0 (0,0)	0 (0,0)	0 (0,0)	0 (0,0)	0 (0,0)	0 (0,0)	0 (0,0)	0 (0,0)
1-9	0 (0,0)	0 (0,0)	0 (0,0)	0 (0,0)	0 (0,0)	0 (0,0)	0 (0,0)	0 (0,0)	0 (0,0)	0 (0,0)	0 (0,0)
None	0 (0,0)	0 (0,0)	0 (0,0)	0 (0,0)	0 (0,0)	0 (0,0)	0 (0,0)	0 (0,0)	0 (0,0)	0 (0,0)	0 (0,0)
Livestock											
≥1	0 (0,0)	0 (0,0)	0 (0,0)	0 (0,0)	0 (0,0)	0 (0,0)	0 (0,0)	0 (0,0)	0 (0,0)	0 (0,0)	0 (0,0)
None	0 (0,0)	0 (0,0)	0 (0,0)	0 (0,0)	0 (0,0)	0 (0,0)	0 (0,0)	0 (0,0)	0 (0,0)	0 (0,0)	0 (0,0)
Cemented walls											
Yes	0 (0,0)	0 (0,0)	0 (0,0)	0 (0,0)	0 (0,0)	0 (0,0)	0 (0,0)	0 (0,0)	0 (0,0)	0 (0,0)	0 (0,0)
No	0 (0,0)	0 (0,0)	0 (0,0)	0 (0,0)	0 (0,0)	0 (0,0)	0 (0,0)	0 (0,0)	0 (0,0)	0 (0,0)	0 (0,0)
MUAC (cm)											
>21.5	0 (0,0)	0 (0,0)	0 (0,0)	0 (0,0)	0 (0,0)	0 (0,0)	0 (0,0)	0 (0,0)	0 (0,0)	0 (0,0)	0 (0,0)
≤21.5	0 (0,0)	0 (0,0)	0 (0,0)	0 (0,0)	0 (0,0)	0 (0,0)	0 (0,0)	0 (0,0)	0 (0,0)	0 (0,0)	0 (0,0)
Literate											
Yes	0 (0,0)	0 (0,0)	0 (0,0)	0 (0,0)	0 (0,0)	0 (0,0)	0 (0,0)	0 (0,0)	0 (0,0)	0 (0,0)	0 (0,0)
No	0 (0,0)	0 (0,0)	0 (0,0)	0 (0,0)	0 (0,0)	0 (0,0)	0 (0,0)	0 (0,0)	0 (0,0)	0 (0,0)	0 (0,0)

F. Seasonal fruits during their in and out-of-season periods														
Characteristics	In-season							Out-of-season						
	Mango	Jackfruit	Guava	Orange/ tangerine	Ripe papaya	Apple	Pineapple	Mango	Jackfruit	Guava	Orange/ tangerine	Ripe papaya	Apple	Pineapple
Caste														
Hindu-High	0 (0,2)	0 (0,0)	0 (0,3)	0 (0,1)	0 (0,1)	0 (0,1)	0 (0,0)	0 (0,0)	0 (0,0)	0 (0,0)	0 (0,0)	0 (0,0)	0 (0,0)	0 (0,0)
Hindu-Low	0 (0,1)	0 (0,0)	0 (0,2)	0 (0,0)	0 (0,0)	0 (0,0)	0 (0,0)	0 (0,0)	0 (0,0)	0 (0,0)	0 (0,0)	0 (0,0)	0 (0,0)	0 (0,0)
Non-Hindu	0 (0,1.5)	0 (0,0)	0 (0,3)	0 (0,0)	0 (0,0)	0 (0,0)	0 (0,0)	0 (0,0)	0 (0,0)	0 (0,0)	0 (0,0)	0 (0,0)	0 (0,0)	0 (0,0)
Land in *kattha*														
≥10	0 (0,1)	0 (0,0)	0 (0,2)	0 (0,0)	0 (0,0)	0 (0,0)	0 (0,0)	0 (0,0)	0 (0,0)	0 (0,0)	0 (0,0)	0 (0,0)	0 (0,0)	0 (0,0)
1-9	0 (0,1)	0 (0,0)	0 (0,2)	0 (0,0)	0 (0,0)	0 (0,0)	0 (0,0)	0 (0,0)	0 (0,0)	0 (0,0)	0 (0,0)	0 (0,0)	0 (0,0)	0 (0,0)
None	0 (0,1)	0 (0,0)	0 (0,2)	0 (0,0)	0 (0,0)	0 (0,0)	0 (0,0)	0 (0,0)	0 (0,0)	0 (0,0)	0 (0,0)	0 (0,0)	0 (0,0)	0 (0,0)
Livestock														
≥1	0 (0,1)	0 (0,0)	0 (0,2)	0 (0,0)	0 (0,0)	0 (0,0)	0 (0,0)	0 (0,0)	0 (0,0)	0 (0,0)	0 (0,0)	0 (0,0)	0 (0,0)	0 (0,0)
None	0 (0,2)	0 (0,0)	0 (0,2)	0 (0,0)	0 (0,0)	0 (0,0)	0 (0,0)	0 (0,0)	0 (0,0)	0 (0,0)	0 (0,0)	0 (0,0)	0 (0,0)	0 (0,0)
Cemented walls														
Yes	0 (0,2)	0 (0,0)	0 (0,3)	0 (0,0)	0 (0,1)	0 (0,0)	0 (0,0)	0 (0,0)	0 (0,0)	0 (0,0)	0 (0,0)	0 (0,0)	0 (0,0)	0 (0,0)
No	0 (0,1)	0 (0,0)	0 (0,2)	0 (0,0)	0 (0,0)	0 (0,0)	0 (0,0)	0 (0,0)	0 (0,0)	0 (0,0)	0 (0,0)	0 (0,0)	0 (0,0)	0 (0,0)
MUAC (cm)														
>21.5	0 (0,1)	0 (0,0)	0 (0,2)	0 (0,0)	0 (0,0)	0 (0,0)	0 (0,0)	0 (0,0)	0 (0,0)	0 (0,0)	0 (0,0)	0 (0,0)	0 (0,0)	0 (0,0)
≤21.5	0 (0,1)	0 (0,0)	0 (0,2)	0 (0,0)	0 (0,0)	0 (0,0)	0 (0,0)	0 (0,0)	0 (0,0)	0 (0,0)	0 (0,0)	0 (0,0)	0 (0,0)	0 (0,0)
Literate														
Yes	0 (0,2)	0 (0,0)	0 (0,3)	0 (0,1)	0 (0,1)	0 (0,1)	0 (0,0)	0 (0,0)	0 (0,0)	0 (0,0)	0 (0,0)	0 (0,0)	0 (0,0)	0 (0,0)
No	0 (0,1)	0 (0,0)	0 (0,2)	0 (0,0)	0 (0,0)	0 (0,0)	0 (0,0)	0 (0,0)	0 (0,0)	0 (0,0)	0 (0,0)	0 (0,0)	0 (0,0)	0 (0,0)
